# The Neuroprotective Effect of *Zingiber cassumunar* Roxb. Extract on LPS-Induced Neuronal Cell Loss and Astroglial Activation within the Hippocampus

**DOI:** 10.1155/2020/4259316

**Published:** 2020-05-25

**Authors:** Ratchaniporn Kongsui, Napatr Sriraksa, Sitthisak Thongrong

**Affiliations:** ^1^Division of Physiology, School of Medical Sciences, University of Phayao, Phayao 56000, Thailand; ^2^Division of Anatomy, School of Medical Sciences, University of Phayao, Phayao 56000, Thailand

## Abstract

The systemic administration of lipopolysaccharide (LPS) has been recognized to induce neuroinflammation which plays a significant role in the pathogenesis of neurodegenerative diseases such as Alzheimer's disease and Parkinson's disease. In this study, we aimed to determine the protective effect of *Zingiber cassumunar* (*Z*. *cassumunar*) or Phlai (in Thai) against LPS-induced neuronal cell loss and the upregulation of glial fibrillary acidic protein (GFAP) of astrocytes in the hippocampus. Adult male Wistar rats were orally administered with *Z*. *cassumunar* extract at various doses (50, 100, and 200 mg/kg body weight) for 14 days before a single injection of LPS (250 *μ*g/kg/i.p.). The results indicated that LPS-treated animals exhibited neuronal cell loss and the activation of astrocytes and also increased proinflammatory cytokine interleukin- (IL-) 1*β* in the hippocampus. Pretreatment with *Z*. *cassumunar* markedly reduced neuronal cell loss in the hippocampus. In addition, *Z*. *cassumunar* extract at a dose of 200 mg/kg BW significantly suppressed the inflammatory response by reducing the expression of GFAP and IL-1*ß* in the hippocampus. Therefore, the results suggested that *Z*. *cassumunar* extract might be valuable as a neuroprotective agent in neuroinflammation-induced brain damage. However, further investigations are essential to validate the possible active ingredients and mechanisms of its neuroprotective effect.

## 1. Introduction

Neuroinflammation plays an important role in the pathogenesis of various neurodegenerative diseases including Alzheimer's disease (AD), Parkinson's disease (PD), Huntington's disease, and dementia [1–3]. In addition, neuroinflammation has implicated in other brain pathological conditions including depression and cerebral ischemia [4]. In the central nervous system (CNS), microglia and astrocytes are displayed as immune effector cells that are involved in neuroinflammation [5, 6]. Activation of the peripheral immune system primes increasing of cytokine levels that are transported into the CNS-stimulating astrocytes and microglial cells [7]. Several studies have provided the evidence that the activation of these glial cells in responding to immune stimuli produces various inflammatory cytokines such as tumor necrosis factor- (TNF-) *α*, interleukin- (IL-) 1*β*, and nitric oxide (NO) that trigger neuronal damage [8–10] and cognitive impairment [11, 12]. Lipopolysaccharide (LPS) is an endotoxin, which is a component of the outer cell wall of gram-negative bacteria, and widely used for the experimental model of neuroinflammation associated with neurodegeneration [13, 14]. Preclinical studies have revealed that intraperitoneal injection of LPS can result in the increased production of inflammatory mediators in the brain, leading to neuronal loss and learning and memory impairments [11, 15–17]. Moreover, LPS also induces sickness-like behaviors including weight loss, decreased locomotion, and increased anxiety [18–20].

To date, there is a growing interest in the neuroprotective effect of herbal medicinal plants mainly against brain damage such as ginger [21, 22], ginkgo [23], ginseng [24], and curcumin [25, 26]. *Zingiber cassumunar* (*Z*. *cassumunar*), a plant in the Zingiberaceae family, is commonly known as “Phlai” in Thai. This medicinal plant consisting of underground rhizomes that have been used for traditional treatment to treat various illnesses such as inflammation, muscle and joint problems, skin diseases, and wound healing [27]. *Z*. *cassumunar* has been reported to have biological properties including anti-inflammation [28], antioxidant [29], and antiallergic [30]. However, none have investigated the neuroprotective effect of *Z*. *cassumunar* on animal models of neuroinflammation induced by LPS. Therefore, the present study was designed to examine whether pretreatment with *Z*. *cassumunar* extract would be effective for neuroprotection against LPS-induced neuronal cell loss by inhibiting astroglial activation in the hippocampus.

## 2. Materials and Methods

### 2.1. Animals

Adult male Wistar rats aged 8 weeks were obtained from the Nomura Siam International Co., Ltd. (Bangkok, Thailand) and were allowed a week for acclimatization prior to the commencement of experimentation. All animals were housed and maintained on a 12 : 12 h light-dark cycle in a constant temperature (21 ± 1°C) and humidity room with food and water available ad libitum. All procedures were approved by the Ethics Committee of the Laboratory Animal Research Center, University of Phayao (approval no. 60 01 04 022).

### 2.2. Plant Materials and Preparation

The fresh rhizomes of *Z*. *cassumunar* were bought from a local market in Muang District, Phayao Province, Thailand. The extraction was conducted using the method described previously by Wong-a-nan et al. [31]. Briefly, the samples were cleaned and cut to small pieces then dried with hot air oven at 60°C for 48 h. Dried samples (4.5 kg of *Z*. *cassumunar*) were ground and extracted with 95% *v*/*v* ethanol by maceration. The ethanol was removed from the extracts under vacuum condition to yield ethanol crude extracts.

### 2.3. The Experimental Protocols

**Figure 1:**
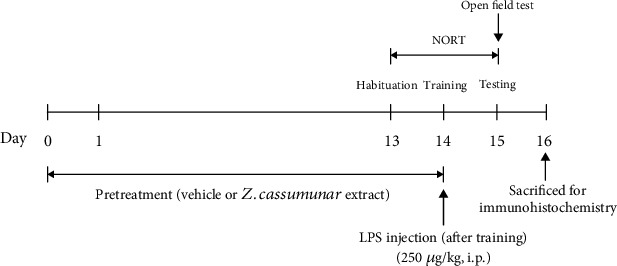
Experimental protocol and schedule of animal tests. NORT: novel object recognition test; LPS: lipopolysaccharide.

All animals were randomly divided into five experimental groups (*n* = 6/group) as follows: (1) the control group, animals received only vehicle; (2) LPS+vehicle; (3) LPS+*Z*. *cassumunar* 50 mg/kg body weight (BW); (4) LPS+*Z*. *cassumunar* 100 mg/kg BW, and (5) LPS+*Z*. *cassumunar* 200 mg/kg BW. Animals were given 1% of carboxymethyl cellulose (CMC) used as vehicle or the extract of *Z*. *cassumunar* (at doses of 50, 100, or 200 mg/kg BW) via oral gavage once daily for 14 days before LPS injection. LPS from *Escherichia coli* (strain 0111:B4, Sigma-Aldrich, St. Louis, MO, USA) was dissolved in 0.9% sterile saline. After training for the object recognition test (day 14), the animals received immediately a single intraperitoneal injection of LPS (250 *μ*g/kg). The dose of LPS used was chosen based on the previous study [32]. For all animals, body weight, food, and water consumption both before and 24 h post-LPS administration to confirm the efficacy of LPS-induced sickness behavior were monitored. The behavioral tests were also measured 24 h before and following the injection of LPS. Twenty-four hours after the behavioral tests, all animals were sacrificed for immunohistochemical study (Figure 1).

### 2.4. Tissue Processing and Immunohistochemistry

All animals were deeply anesthetized via intraperitoneal injection of sodium pentobarbital (70 mg/kg BW) and transcardially perfused with ice cold 0.1 M PBS for 5 min followed by ice cold 4% paraformaldehyde (pH 7.4) for 15 min. Brains were removed and postfixed overnight with the same fixative. Then, brains were placed into 12.5% sucrose for cryoprotection. Brains were sectioned into 30 *μ*m using a cryostat microtome (AST500, Amos Scientific) and stored in an antifreeze solution (4°C) for immunoperoxidase labeling. Coronal sections were rinsed and incubated in 3% H_2_O_2_ and followed by 3% normal horse serum for nonspecific blocking. Then, sections were incubated with primary antibody; mouse anti-glial fibrillary acidic protein (GFAP, 1 : 1000, Millipore, USA) or rabbit anti-IL-1*β* (IL-1*β*, 1 : 100; Millipore, USA) at 4°C overnight. Sections were washed in 0.1 M PBS for 30 min and incubated for 2 h at room temperature with biotinylated donkey anti-mouse secondary antibody or biotinylated donkey anti-rabbit (1 : 500, Jackson Immunoresearch, USA). Sections were rinsed and followed by an hour incubation in 0.1% extravidin peroxidase (1 : 1000, Sigma-Aldrich, St. Louis, MO, USA), and then rinsed again. Immunolabeling was developed using a nickel-enhanced 3, 3′-diaminobenzidine (DAB) reaction. Finally, sections were washed and then mounted on positive slides, dehydrated using graded alcohols (70, 95, and 100% for 1 min each), cleared in xylene for 3 min, and cover slipped using DPX mountant (Fisher Scientific, UK). For cresyl violet staining, sections were stained with 0.2% cresyl violet (Sigma-Aldrich, St. Louis, MO, USA), dehydrated through an ethanol series (70, 95, and 100% 2x), cleared in xylene, and cover slipped using DPX.

### 2.5. Thresholding and Cell Count Analysis

Hippocampal images (-3.14 mm from Bregma) were captured at 20x using a bright-field microscope (Olympus). The immunoreactivity signal was determined using ImageJ software. The images were cropped into 4 subregions of the hippocampus including CA1, CA2, CA3, and the dentate gyrus (DG). These cropped regions were thresholded, and the data were presented as the percentage of thresholded area. To determine the density of the neuron, 40x images of subregions of the hippocampus were exhaustive manual counts by using SXView program.

### 2.6. Novel Object Recognition Test (NORT)

The novel object recognition test was performed in a black plastic apparatus (45 cm width × 65 cm length × 45 cm height) with a constant light condition (40 lux). The test consisted of 5 min three sessions: habituation, training, and testing as previously described [33]. All animals were habituated to the empty apparatus for 5 min and then returned to their home cage. On the training day, animals were placed into the apparatus to explore two familiar objects (object A) which was placed in the symmetrical corner of the apparatus for 5 min. Twenty-four hours after training, the animals were allowed to investigate the objects in which one of the familiar objects was replaced by a novel object (B). The exploration time identified as pointing the nose to the object at a distance ≤2 cm was recorded. The discrimination index was determined for recognition memory, calculated as the difference of time spent exploring the novel (B) and the familiar (A) object multiplied by100 divided by the sum of time spent exploring the novel object (B) and the familiar object (A). The box and the objects were cleaned thoroughly with 70% ethanol after each animal finished to avoid perturbation of the animals due to urine and feces. Animals showing a total exploration time < 10 s were excluded. Sitting or climbing on the object was not measured as exploration.

### 2.7. Open Field

To determine the locomotor activity, the animals were placed in the center of 45 × 65 × 45 cm apparatus following the novel object recognition task. The floor of the apparatus was divided into 16 squares. The animals were allowed to explore the field for 5 min. The locomotor activity was assessed by testing the number of lines crossings over a 5 min period [34].

### 2.8. Statistical Analysis

All data were analyzed using GraphPad Prism 8 (GraphPad Software, USA). Data were expressed as the mean ± S.E.M. Statistical analysis was performed using one-way ANOVA, followed by Tukey's post hoc test for multiple comparisons. *p* < 0.05 was considered statistically significant.

## 3. Results

### 3.1. Effect of *Z*. *cassumunar* on Body Weight, Food Intake, and Locomotor Activity in LPS-Induced Sickness Behavior

**Figure 2:**
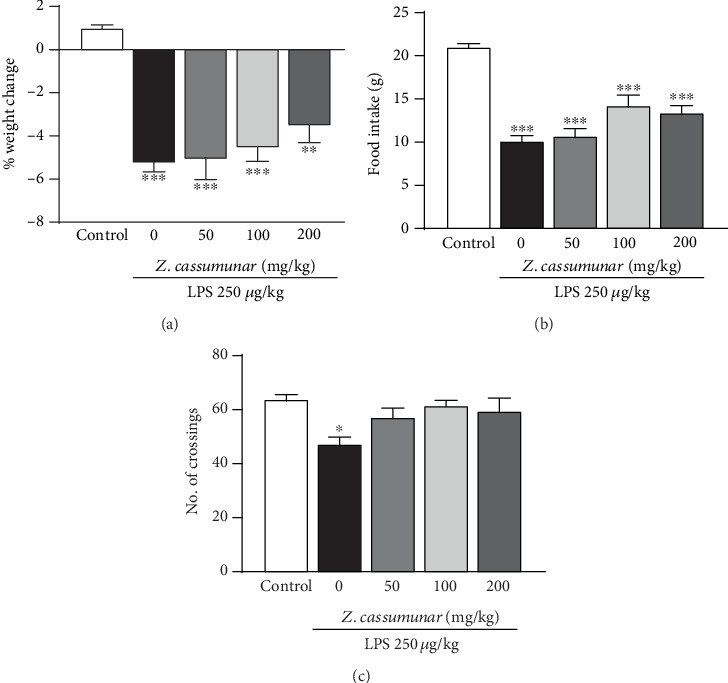
Effect of *Z*. *cassumunar* on body weight, food intake, and locomotor activity. LPS-treated animals showed a significant reduction of body weight (a), food intake, (b) and the number of line crossings in the open field (c). Treatments with *Z*. *cassumunar* did not affect body weight, food intake, and locomotor activity. Data were expressed as the mean ± SEM (*n* = 6). ^∗^*p* < 0.05, ^∗∗^*p* < 0.01, and ^∗∗∗^*p* < 0.001 vs. the control group.

The administration of LPS is a well-characterized model for neuroinflammation. Twenty-four hours after the injection, the body weight, food intake, and locomotor activity were determined. In this study, we found that a single injection of LPS at a dose of 250 *μ*g/kg induced sickness-like behavior. LPS-treated animals demonstrated a significant reduction of body weight (Figure 2(a)) and food intake (Figure 2(b)) compared to the control animals (*p* < 0.001). The locomotor activity was determined by testing the number of crossings in the open field test. It was reported that LPS also significantly disturbed the locomotor activity of animals after the injection when compared to the control group (*p* < 0.05) (Figure 2(c)). However, the extract of *Z*. *cassumunar* at various doses (50, 100, and 200 mg/kg) was not able to prevent LPS-induced sickness-like behavior.

### 3.2. Effect of *Z*. *cassumunar* on Recognition Memory

**Figure 3:**
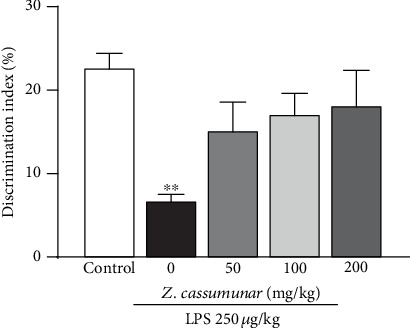
Effect of *Z*. *cassumunar* on the recognition memory in the novel object recognition test. LPS-treated animals showed the impairment of recognition memory by a reduction of the discrimination index. The extract of *Z*. *cassumunar* did not improve memory impairment induced by LPS. Data were expressed as the mean ± SEM (*n* = 6). ^∗∗^*p* < 0.01 vs. the control group.

In this part, the novel object recognition test was used to determine whether *Z*. *cassumunar* could reverse LPS-induced recognition impairment (Figure 3). The animals that received LPS showed memory impairment in long-term memory retention (delay 24 h) by decreasing the discrimination index compared to the control animals (*p* < 0.01). However, all doses of *Z*. *cassumunar* extract tended to increase the discrimination index (*p* = 0.079, *p* = 0.062, and *p* = 0.058, respectively) but did not improve recognition impairment induced by LPS, as observed by the fact that there were no significant differences between groups in exploration time with the novel object.

### 3.3. Effect of *Z*. *cassumunar* on Neuronal Density in the Hippocampus

**Figure 4:**
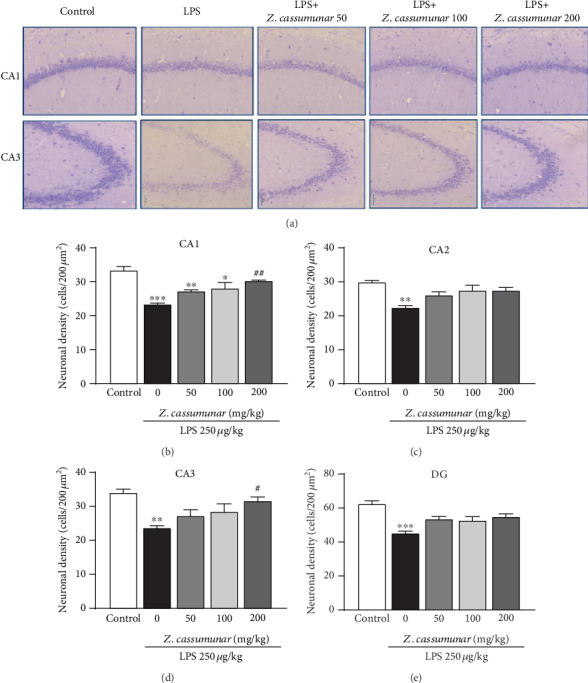
Effect of *Z*. *cassumunar* on neuronal density in the hippocampus. Representative images of CA1 and CA3 histologically stained with cresyl violet at 20x magnification (a). Graphs showed neuronal density in the hippocampus (b–e). The extract of *Z*. *cassumunar* significantly reversed LPS-induced neuronal cell loss in CA1 and CA3. Data were expressed as the mean ± SEM (*n* = 6). ^∗^*p* < 0.05, ^∗∗^*p* < 0.01, and ^∗∗∗^*p* < 0.001 vs. the control group; ^#^*p* < 0.05, ^##^*p* < 0.01 vs. the vehicle-treated LPS group.

We also investigated the effect of *Z*. *cassumunar* on neuronal density was detected by the cresyl violet staining of Nissl bodies (Figure 4(a)). The data demonstrated that LPS administration significantly decreased the neuronal density in all subregions of the hippocampus. Interestingly, the extract of *Z*. *cassumunar* at a dose of 200 mg/kg significantly prevented the neuronal cell loss in CA1 and CA3 regions of the hippocampus compared to the LPS+vehicle group (CA1; *p* < 0.01, Figure 4(b) and CA3; *p* <0.05 Figure 4(d)).

### 3.4. Effect of *Z*. *cassumunar* on the Immunoreactivity of GFAP in the Hippocampus

**Figure 5:**
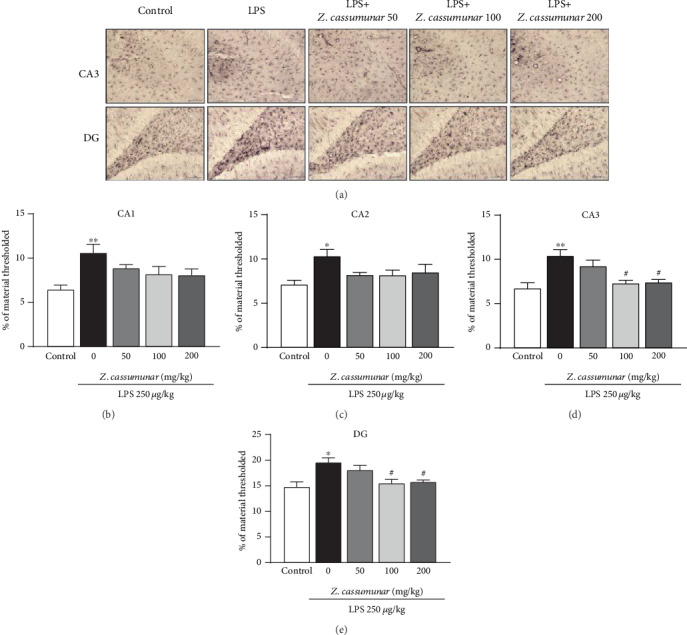
Effect of *Z*. *cassumunar* on the immunoreactivity of GFAP in the hippocampus. Representative images of immunohistochemical staining of GFAP in CA3 and DG (a). Graphs demonstrated the GFAP material thresholded in all subregions of the hippocampus (b–e). LPS-treated animals with *Z*. *cassumunar* showed a remarkable decrease in the levels of GFAP expression in CA3 and DG. Data were expressed as the mean ± SEM (*n* = 6). ^∗^*p* < 0.05, ^∗∗^*p* < 0.01 vs. the control group; ^#^*p* < 0.05 vs. the vehicle-treated LPS group.

In response to LPS-induced neuroinflammation, astrocyte activation was implicated as one of the primary responders to the inflammatory process associated with brain damage. We further examined the effect of *Z*. *cassumunar* on the expression of GFAP (a marker of astrocyte activation) in the hippocampus. As the results show (Figure 5), immunohistochemical labeling of GFAP assessed via threshold analysis demonstrated a significant upregulation of GFAP expression in all regions of the hippocampus following LPS injection (Figures 5(b)–5(e)). However, we found that pretreatment with *Z*. *cassumunar* extract at medium and high doses (100 and 200 mg/kg) markedly attenuated LPS-induced overexpression of astrocytes particularly in the CA3 and DG regions of the hippocampus compared with the vehicle LPS-treated group (*p* < 0.05).

### 3.5. Effect of *Z*. *cassumunar* on the Immunoreactivity of IL-1*ß* in the Hippocampus

**Figure 6:**
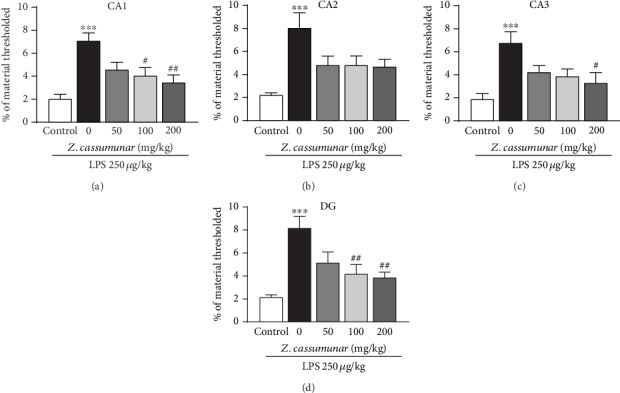
Effect of *Z*. *cassumunar* on the immunoreactivity of IL-1*ß* in the hippocampus. Graphs demonstrated the IL-1*ß* material thresholded in all subregions of the hippocampus (a–d). LPS-treated animals with *Z*. *cassumunar* showed a remarkable decrease in the levels of IL-1*ß* expression in CA1, CA3, and DG. Data were expressed as the mean ± SEM (*n* = 6). ^∗^*p* < 0.05, ^∗∗^*p* < 0.01 vs. the control group; ^#^*p* < 0.05, ^##^*p* < 0.01 vs. the vehicle-treated LPS group.

As IL-1*β* is an important proinflammatory cytokine in the brain, we next investigated whether *Z*. *cassumunar* extract could modulate IL-1*β* expression within the hippocampus by using immunohistochemistry staining. Treatment with LPS significantly increased the immunoreactivity of IL-1*β* in all subregions of the hippocampus when compared to the control group (*p* < 0.001; Figure 6). Pretreatment of *Z*. *cassumunar* at a dose of 100 mg/kg markedly decreased the expression of IL-1*β* in CA1 and DG of the hippocampus (*p* < 0.05, *p* < 0.01; Figures 6(a) and 6(d), respectively). Additionally, the extract of *Z*. *cassumunar* at a dose of 200 mg/kg significantly reduced the upregulation of IL-1*β* in CA1, CA3, and DG of the hippocampus (*p* < 0.01, *p* < 0.05, and *p* < 0.01; Figures 6(a), 6(c), and 6(d), respectively).

## 4. Discussion

In the current study, our results suggested for the first time that pretreatment with *Z*. *cassumunar* extract could prevent neuronal cell loss via suppressing the activation of astrocytes and IL-1*ß* in the hippocampus following the administration of LPS. LPS is a potent inducer of the inflammatory process by triggering of glial cells mainly through Toll-like receptor 4 (TLR4), which in turn produces proinflammatory cytokines, reactive oxygen species (ROS), and NO [35, 36], leading to various neurodegenerative diseases [11, 15]. Numerous studies have reported that the injection of LPS also produced memory deficit and sickness-like behavior including decreased locomotor activity, anorexia, body weight loss, increased anxiety, and somnolence [11, 18, 19] which are considered to be very similar to the clinically important symptoms of neurodegenerative diseases in humans. Therefore, the administration of LPS is frequently used to study neuroinflammation-related neurodegenerative diseases in animals.

The findings demonstrated that LPS-treated rats significantly displayed behavioral changes including decrease in body weight, food consumption, and exploratory behavior following the injection of LPS, suggesting that LPS can induce obvious sickness-like behavior in rats. However, the treatment with *Z*. *cassumunar* extract did not reverse the LPS-induced sickness-like behavior. As previously reported, the increase in levels of proinflammatory cytokines such as IL-1*α*, IL-1*ß*, TNF, and IL-6 induced by LPS can result in sickness behavior [12, 37]. Inflammatory cytokines within the brain can also contribute to excessive generation of free radicals correlated with behavioral impairment [38]. The sickness-like behavior is also involved in a sustained cellular reactivity in various regions of the brain including the amygdala, the hippocampus, and the hypothalamus [39]. In the present study, we only measured the expression of IL-1*ß* in the hippocampus and did not measure the other proinflammatory mediators such as TNF, IL-6, and NO which are associated with the alterations of sickness-like behavior. In addition, the onset of sickness behavior due to a high dose of LPS (250 *μ*g/kg) used in this study might be a crucial factor for an incomplete restoration of behavior by the treatment of extract. Thus, it is assumed that the potential effect of *Z*. *cassumunar* extract at the high dose (200 mg/kg BW) was insufficient to improve LPS-induced sickness-like behavior via modulating of proinflammatory cytokines IL-1*ß*.

Moreover, our results revealed that LPS also has the potential to induce recognition deficit by decreasing the discrimination index in the novel object recognition test. However, pretreatment with *Z*. *cassumunar* at various doses was not able to significantly improve recognition memory after a 24 h retention interval. Previous studies have reported that the animals usually explore the new objects longer than the familiar objects when the interval between the training trial and the test trial is 1 h or less. The animals cannot recognize the familiar and the new objects during longer retention [40, 41]. However, our result reported that the cognition impairment in long-term memory is not reversed by *Z*. *cassumunar* pretreatment which may relate to the incomplete recovery of the hippocampus damage from LPS-induction.

As neuroinflammation, the activation of astrocytes is stated to be a key factor in the pathophysiology of neurodegeneration [9, 42]. There is evidence that the increased expression of GFAP, a vital protein in the astrocyte cytoskeleton, represents astrocyte activation [43]. Additionally, it has also been suggested that GFAP is important for the interaction of astrocytes and neurons and plays a crucial role in regulating synaptic function in the CNS [44]. Furthermore, previous studies have revealed that the modulation of astroglial activation can regulate neuronal survival and cognition [45, 46]. Therefore, in this study, we focused on the protective effect of *Z*. *cassumunar* particularly the alterations of neurons and astrocytes in the hippocampus associated with cognitive function. The present data demonstrated that the peripheral LPS administration significantly decreased the neuronal density in all subregions of the hippocampus when compared to the control group. *Z*. *cassumunar* extract at a dose of 200 mg/kg was effective against the neuronal cell loss in the CA1 and CA3 regions of the hippocampus. As previously reported, the hippocampus can be affected by several pathological conditions such as ischemia, inflammation, and oxidative stress [47]. It has been also suggested that CA1 and CA3 importantly contribute a crucial role in the memory process [48, 49]. Upon activation, astrocytes generally alter their morphology for being hypertrophic and express an increased expression of GFAP [50]. This study reported that LPS induction significantly increased the immunoreactivity of GFAP in the hippocampus, while pretreatment with *Z*. *cassumunar* (100 and 200 mg/kg) was able to suppress the expression of GFAP in the hippocampus particularly in CA3 and DG. Several investigations have suggested that increased expression of GFAP is associated with the excessive production of proinflammatory substances such as IL-1*β*, TNF-*α*, and NO [51, 52]. IL-1*β* is a pleiotropic cytokine family, which is involved in acute neuroinflammation-related diseases [53]. IL-1*β* is mainly produced by microglia and astrocytes [54, 55]. The present study showed that LPS injection significantly elevated the expression of proinflammatory cytokine IL-1*β* in the hippocampus, while *Z*. *cassumunar* extract at a dose of 200 mg/kg BW significantly reduced the expression of IL-1*β* in CA1, CA3, and DG. Thus, the results indicated that pretreatment with *Z*. *cassumunar* can increase neuronal density through the inhibition of the expression of GFAP and IL-1*β* in the hippocampus. The possible mechanisms of reduced neuronal cell loss might be involved in anti-inflammatory and antioxidant activities of the extract by suppressing astroglial activation-induced IL-1*β* production. Evidence from a previous study has reported that isolated phenylbutanoids from *Z*. *cassumunar* rhizomes have anti-inflammatory activity by the inhibition of LPS-induced NO production in mouse peritoneal macrophages [56]. Additionally, phenylbutanoid, E-4-(3′,4′-dimethoxyphenyl)but-3-en-1-ol, from *Z*. *cassumunar* exhibited anti-inflammatory effect in rats. However, isolated chemical active ingredients from *Z*. *cassumunar* have not been determined for neuroprotective activity in the animal models. Considering neuroprotective effects of active compound from a medicinal plant that is closely related to *Z*. *cassumunar*, gingerol from *Z*. *officinale* exerts neuroprotective effects against oxidative stress-related neurodegeneration [57, 58]. Recently, it has been reported that gingerol decreases LPS-induced neuroinflammation and cognitive impairment through inhibiting astrocyte overactivation in rats [59].

To our knowledge, this is the first study demonstrating the neuroprotective effects of *Z*. *cassumunar* extract on the neuronal cell loss via suppressing the overactivation of astrocytes. The important limitations of this study are the lack of the identification of bioactive ingredients and relevant molecular mechanisms of beneficial effects. However, since we now demonstrated the neuroprotective activity of *Z. cassumunar* crude extract against neuronal damage and neuroinflammation, we recommend that further investigations of the main bioactive compounds are required to provide better understanding concerning the neuroprotective effects.

## 5. Conclusions

In conclusion, intraperitoneal injection of LPS-induced neuronal cell loss caused an inflammatory response by activating the astrocytes and increasing the expression of proinflammatory cytokine IL-1*β* in the hippocampus. Pretreatment with *Z*. *cassumunar* crude extract attenuated LPS-induced neuronal cell loss by reducing the expression of GFAP and IL-1*ß* in the hippocampus. Therefore, the present study suggested that *Z*. *cassumunar* crude extract might be a potential neuroprotective agent for the treatment of LPS-induced neurodegenerative diseases.

## Data Availability

The data used to support the findings of this study are included within the article.
